# Supplementary material for: Adaptation of a short and universal learning self-efficacy scale for clinical skills in Turkish
Source: PLoS One. 2022 Nov 10;17(11):e0275672. doi: 10.1371/journal.pone.0275672 (PMC9648743; doi:10.1371/journal.pone.0275672)
Supplement: Attachment — Submitted filename: Adaptation L-SES.docx [file pone.0275672.s001.docx]

**Adaptation of a Short and Universal Learning Self Efficacy Scale for Clinical Skills in Turkish**

**Abstract**

In this study, we aimed to adopt linguistic equivalence, validity, and reliability study of a self-efficacy scale for clinical skills (L-SES) developed by Kang, Chang [1]. (No ref in the abstract) they should add in the text (Introduction part and then re number the references accordingly

**Introduction**

Considering that most students tend to be overconfident about their capabilitie~~s [4], (~~ No need to add as reference , this is a general statement )

The purpose of this study is to adapt the “Short and Universal Learning Self-Efficacy Scale for Clinical Skills” questionnaire into the Turkish language/culture and applying validity and reliability analyses

**Materials and Methods**

~~The aim of this research was to carry out a validity and reliability study of the short universal Learning Self-Efficacy Scale developed for clinical skills by adapting it to the Turkish Language and culture~~. It was mentioned in the Introduction section so need to repeat in this section

The name and surname information were not requested from the participants, only age, gender, ~~nationality,~~ and period information. Why authors need nationality to be included in the demographic section of the questionnaire?

**Results**

What authors do mean by putting Item 1 Item 2 Item 3	-----------------Item 15

Authors have gathered data on the previously developed questionnaire that focused on self-efficacy. They didn’t mention for what clinical skills they gathered the data, e.g., history taking, GIT examination, Cranial nerves, etc. they must mention what were the clinical skills in year 1 and Year 3 they collected the data to perceive the perception of efficacy.

They have written Item 1 to Item 15 what are these items, to understand this article, some one must read first

*Development of a short and universal learning self-efficacy scale for clinical skills*

*Yi-No KangID, Chun-Hao Chang, Chih-Chin Kao, Chien-Yu Chen, ChienChih Wu,*
